# Bone disease in patients with chronic kidney disease under conservative management

**DOI:** 10.1590/S1516-31802005000200010

**Published:** 2005-03-02

**Authors:** Carlos Perez Gomes, Maria Inês Barreto Silva, Maria Eugênia Leite Duarte, David Dorigo, Carla Cavalheiro da Silva Lemos, Rachel Bregman

**Keywords:** Renal osteodystrophy, Kidney diseases, Chronic kidney failure, Renal dialysis, Bone resorption, Osteodistrofia renal, Nefropatias, Insuficiência renal crônica, Hemodiálise, Reabsorção óssea

## Abstract

**CONTEXT AND OBJECTIVE:**

Few studies have focused on bone disease in patients with chronic kidney disease under conservative treatment. The objective was to evaluate bone disease in patients with chronic kidney disease.

**DESIGN AND SETTING:**

Case series, at the Nephrology Division, Hospital Universitário Pedro Ernesto.

**METHODS:**

131 patients with creatinine clearance from 10 to 60 ml/min/1.73 m² were followed up for at least one year. Serum creatinine, albumin, calcium, phosphorus, alkaline phosphatase, total CO_2_ (tCO_2_), intact parathyroid hormone (iPTH), and alkaline phosphatase were measured. Creatinine clearance was calculated from 24-hour urine creatinine measurements and protein ingestion estimates from urea assays.

**RESULTS:**

Patients presenting creatinine clearance < 30 ml/min/1.73 m² had higher iPTH values, but normal serum levels for calcium, phosphorus, alkaline phosphatase and tCO_2_. Patients presenting iPTH values of twice the normal upper limit (144 pg/ml) showed lower tCO_2_ values. Bone alkaline phosphatase was evaluated in 37 patients with creatinine clearance < 30 ml/min/1.73 m², showing correlation with alkaline phosphatase but not with parathyroid hormone. Bone biopsy on nine patients with creatinine clearance < 30 ml/min/1.73 m² and iPTH > 144 pg/ml showed osteitis fibrosa (4), mild lesion (4) and high turnover (1).

**CONCLUSION:**

The present data suggest the importance of early control for iPTH and metabolic acidosis, among patients under conservative management for chronic kidney disease, in order to prevent complications related to bone disease.

## RESUMO

**CONTEXTO E OBJETIVO:**

A doença óssea em pacientes com doença renal crônica sob tratamento conservador tem sido pouco estudada. O objetivo do estudo foi avaliar a doença óssea em pacientes com doença renal crônica.

**TIPO DE ESTUDO E LOCAL:**

Estudo de casos, na Disciplina de Nefrologia, Hospital Universitário Pedro Ernesto.

**MÉTODOS:**

Foram avaliados por pelo menos um ano 131 pacientes com *clearance* de creatinina variando de 10 a 60 ml/min/1,73 m^2^. No plasma, foram medidos creatinina, albumina, cálcio, fósforo, fosfatase alcalina, CO_2_ total (tCO_2_), hormônio paratiroidiano intacto (iPTH) e fosfatase alcalina. Da urina de 24 horas, foram avaliadas o *clearance* de creatinina e a ingestão protéica.

**RESULTADOS:**

Pacientes com *clearance* de creatinina < 30 ml/min/1,73 m² apresentaram maior nível de iPTH, apesar de valores normais para.cálcio, fósforo, fosfatase alcalina e tCO_2_. Pacientes com valores de iPTH duas vezes acima do valor superior da normalidade (144 pg/ml) apresentaram menor valor de tCO_2_. Fosfatase alcalina óssea foi avaliada em 37 pacientes com *clearance* de creatinina < 30 ml/min/1,73m², mostrando correlação com a fosfatase alcalina, mas não com o iPTH. Biópsia óssea em nove pacientes com *clearance* de creatinina < 30 ml/min/1,73m² e iPTH > 144 pg/ml mostrou osteíte fibrosa (4), lesão mínima (4) e alto remodelamento (1).

**CONCLUSÃO:**

Os resultados apontam para a importância de um controle precoce do iPTH e da acidose metabólica em pacientes sob tratamento conservador para doença renal crônica, no intuito de prevenir as complicações relacionadas com a doença óssea.

## INTRODUCTION

Chronic kidney disease today is becoming one of the most important public health problems. In Brazil, there are now approximately 60,000 patients undergoing dialytic treatment and it is thought that more than 1,000,000 people have some level of renal dysfunction. This syndrome leads to many long-term complications in different organs (anemia, bone disease, cardiovascular damage and malnutrition) and maintenance of adequate treatment for the population with this set of illnesses imposes a large economic burden. For example, new drugs like the phosphorus binder sevelamer and calcimimetics agents are being used in complicated cases of hyperphosphatemia, in patients under dialysis treatment, but they are expensive. This shows the importance of early diagnosis and treatment, so as to avoid such complications.

Renal osteodystrophy, a common feature among patients with chronic kidney disease, has been widely studied in populations submitted to renal function substitution treatment. When the glomerular filtration rate falls to half of its normal value, approximately 50% of such patients are already presenting histological bone lesions.^1^ There is a lack of data for characterizing the prevalence of renal osteodystrophy among the pre-dialysis population. There are reports suggesting a high prevalence of low-turnover disease,^2,3^ and mixed lesion.^4^ However, high-turnover disease seems to be the main expression of secondary hyperparathyroidism.^1,5^ Bone biopsy is still the gold-standard for the diagnosis of renal osteodystrophy,^1,6,7^ although it is infrequently used at the pre-dialysis phase, when patients are followed by means of serum biochemistry parameters. Among the methods utilized for evaluating parathyroid hormone (PTH), assaying of intact PTH (iPTH) is considered to be the most specific, and this forms a noninvasive method for diagnosing hyperparathyroidism.^3^ Besides iPTH, other markers have been proposed.^8^ Bone alkaline phosphatase is a glycoprotein anchored in the cellular membrane of the osteoblasts that is not filtered by the kidneys or dialysis, and it has been suggested that it would be the best marker for bone disease in chronic kidney disease.^9^ Studies analyzing the bone histology of patients on dialysis have shown good correlation between this marker and osteoblastic activity, and have suggested that it is superior to iPTH and alkaline phosphatase.^10^ This issue has not been well studied in populations under conservative treatment.

The objective of the present study was thus to evaluate the biochemical and hormonal profile related to bone disease, among patients with chronic kidney disease under conservative treatment.

## METHODS

The study group consisted of 131 outpatients who were being followed up by the Nephrology Division of Hospital Pedro Ernesto, Universidade do Estado do Rio de Janeiro. All patients signed an informed consent statement regarding their participation in the study, which had previously been approved by the institution's review board. All patients were followed up by nephrologists and nutrition-ists. The patients’ creatinine clearance ranged from 10 to 60 ml/min/1.73 m^2^. Protein intake had been prescribed, ranging from 0.8 to 1 g/kg/day, and all patients were taking calcium carbonate supplementation. Patients were excluded if they were less than 18 years of age, had had less than one year of follow-up, had parathyroid disease, or were using vitamin D intake, dialysis procedures, hormonal replacement therapy, glucocorticoids, phosphorus binder with aluminum, or drugs that directly interfere in bone metabolism.

Evaluations of serum creatinine, albumin, calcium, phosphorus, total CO_2_ (tCO_2_) and alkaline phosphatase were performed. Urea and creatinine were measured from 24-hour urine. The protein ingestion estimate was calculated using the equation:


Estimated protein ingestion=[(urinary ureax0.466)+(0.031xbody weight)] x6.25/body weight (g of protein/kg/day).

Intact PTH was evaluated by immunochemiluminometric assay (reference values: 12 – 72 pg/ml). Bone alkaline phosphatase was evaluated in 37 patients with creatinine clearance < 30 ml/min/1.73m², by the immunoenzymatic method (reference values: 14.8 – 65.2 U/l).

Nine patients with creatinine clearance < 30 ml/min/1.73 m² and iPTH of twice the highest normal value were submitted to bone biopsy. Patients were given two courses of tetracycline separated by an interval of 12 days. The biopsy was performed four days after the last dose of tetracycline. The bone samples were fixed in 70% ethanol and dehydrated by sequential changes in ascending concentrations of ethanol and xylene and then embedded in methyl methacrylate. For histo-logical analysis, undecalcified sections (5 μm) of cortical and trabecular bone were stained by the Goldner method and by aurintricarboxylic acid for detection of aluminum. Bone remodeling and turnover were investigated on 10-μm unstained sections under fluorescent light.

The histological classification for the skeletal lesions of renal osteodystrophy that was used in this study was as follows: *osteitis fibrosa*: increased remodeling and bone marrow fibrosis; *mild renal osteodystrophy lesion*: slightly increased bone remodeling and no fibrosis; *osteomalacia*: increased osteoid volume and surface, defective mineralization; *adynamic bone disease*: hypocellular bone surfaces, no remodeling; *mixed renal osteodystrophy*: features of both osteitis fibrosa and osteomalacia.^11^

### Statistical analysis

Results are presented as mean ± standard deviation for quantitative variables with symmetrical distribution, and median and range for the variables with asymmetric distribution. Comparisons between two groups were performed using the t test for parametric data, Mann-Whitney test for non-parametric data (iPTH and alkaline phosphatase) and chi-squared test for qualitative variables. Linear correlation analysis was done by means of the Pearson r or Spearman r coefficient, in accordance, respectively, with symmetric or asymmetric distribution of the variables.

## RESULTS

The mean age of the studied population was 61 ± 14 years and 67% were white, 13% black and 20% non-white and non-black. The gender distribution was 54% men and 46% women. The etiologies of the chronic kidney disease were hypertension 39%, diabetes mellitus 23%, unknown 15%, polycystic kidney disease 9%, primary glomerulopathy 6%, chronic pyelonephritis 3% and others 5%.

**Figure 1:**
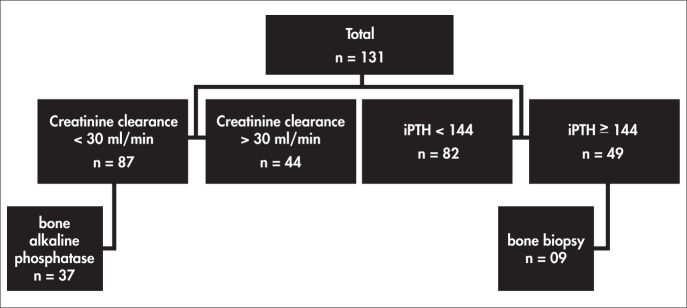
Protocol for 131 patients followed up in an outpatient nephrology service in Rio de Janeiro, Brazil.

The biochemical data were analyzed in two stages (Figure 1). In the first stage (Table 1) patients were divided into two groups according to their creatinine clearance: < 30 ml/min/1.73 m² (n = 87, group I) and > 30 ml/min/1.73 m² (n = 44, group II). There were no differences in age, gender, estimated protein ingestion (0.8 ± 0.3 vs. 0.9 ± 0.3 g/kg/day) and albumin (4.5 ± 0.4 vs. 4.4 ± 0.7 g/dl) between the groups. Group I had a lower value for calcium, and higher values for alkaline phosphatase and iPTH, thus suggesting that these patients’ bone tissue was more severely compromised. Phosphorus and total CO_2_ values were similar in the two groups. It is important to point out that all the parameters analyzed were within their normal ranges.

iPTH = intact parathyroid hormone.

**Table 1:** Profile of 131 patients followed up in an outpatient nephrology service in Rio de Janeiro, Brazil, according to creatinine clearance

	Group I	Group II	p value
	Creatinine clearance	Creatinine clearance	
	≤ 30 ml/min	> 30 ml/min	
	(n = 87)	(n = 44)	
Calcium (mg/dl)	9.2 ± 0.6	9.5 ± 0.6	0.024*
Phosphorus (mg/dl)	4.0 ± 0.7	3.8 ± 0.7	0.13*
Alkaline phosphatase (U/l)	95 (31 - 673)	88 (29 - 165)	0.035**
Total CO_2_ (mEq/l)	22.2 ± 3.9	23.8 ± 3.2	0.02*
iPTH (pg/ml)	136 (1 - 1,435)	84 (5 - 229)	< 0.001**

*iPTH = intact parathyroid hormone;*

* *† test;*

** *Mann-whitney test. Results are expressed as mean ± standard deviation or, for alkaline phosphatase and iPTH, as median (range).*

In the second stage, patients were divided according to their iPTH levels (median) as follows: those with values of up to twice the upper limit for iPTH (group I: iPTH < 144 pg/ml; n = 82) and those above this level (group II: iPTH > 144 pg/ml; n = 49). There was no difference in gender, age, serum albumin (4.5 ± 0.6 vs. 4.4 ± 0.4 g/dl) or estimated protein ingestion (0.8 ± 0.3 g/kg/day, for both) between the groups. Table 2 shows that alkaline phosphatase values were normal, and iPTH was 3-4 times higher in group II than in group I. The group with higher iPTH levels had lower calcium and higher phosphorus values, and also lower total CO_2_ value. In 37 patients with mean age 61 ± 12 years (kidney glomerular filtration rate, GFR, of < 30 ml/min/1.73 m²), bone alkaline phosphatase serum levels were evaluated. This group showed normal mean serum levels for albumin (4.4 ± 0.5 g/l), calcium (9.3 ± 0.6 mg/dl), phosphorus (4.1 ± 0.6 mg/dl) and estimated protein ingestion (EPI) (0.8 ± 0.3 g/kg/day). Mean total CO_2_ was 21.9 ± 3.4 mEq/l, thus indicating metabolic acidosis. The median for iPTH was 137 pg/ml (range: 30 – 752 pg/ml). The median for alkaline phosphatase was 94 U/l (range: 52 – 673 U/I), while the median for bone alkaline phosphatase was 25.8 U/l (range: 8.3 – 50 U/l). A comparison between bone alkaline phosphatase and iPTH showed Spearman r = 0.18. When bone alkaline phosphatase was compared to alkaline phosphatase, a good correlation was observed (Spearman r = 0.69, p< 0.001).

**Table 2:** Profile of 131 studied patients followed up in an outpatient nephrology service in Rio de Janeiro, Brazil, according to iPTH

	Group I	Group II	p value
	iPTH < 144 pg/ml	iPTH ≥ 144 pg/ml	
	(n = 82)	(n = 49)	
Calcium (mg/dl)	9.5 ± 0.6	9.0 ± 0.6	< 0.001*
Phosphorus (mg/dl)	3.9 ± 0.6	4.1 ± 0.7	0.021*
Alkaline phosphatase (U/l)	88 (29 - 673)	97 (31 - 393)	0.196**
Total CO2 (mEq/l)	23.6 ± 3.4	21.3 ± 3.9	0.001*
iPTH (pg/ml)	80 (1 - 142)	273 (147 - 1,435)	< 0.001**

*Results are expressed as mean ± standard deviation or, for alkaline phosphatase and iPTH, as median (range).*

* *= † test;*

** *= Mann-Whitney test; iPTH = intact parathyroid hormone.*

Nine patients with creatinine clearance < 30 ml/min/1.73m² were submitted to bone biopsy, which showed median iPTH of 263 pg/ml (range: 151 – 767 pg/ml), and median bone alkaline phosphatase median of 23.1U/l (range: 8.3 – 50). These values did not present correlation between them. The median alkaline phosphatase of 103 U/l (range: 66 – 180 U/l), albumin (4.3 ± 0.5 g/l), calcium (9.4 ± 0.7 mg/dl), phosphorus (4.6 ± 1.2 mg/dl) and EPI (0.8 ± 0.3 g/kg/day) were normal, although total CO_2_ was low (21.9 ± 3.4 mEq/ l). The histological evaluation showed osteitis fibrosa (n = 4), mild lesion (n = 4) and high turnover alone (n = 1).

## DISCUSSION

This study evaluated outpatients with chronic kidney disease under conservative treatment, and showed their biochemical profiles, focusing on secondary hyperparathyroidism. Bone alkaline phosphatase was also evaluated in order to analyze its utility as a marker for renal osteodystrophy. Additionally, bone biopsies were performed to illustrate bone histology in this population. Recent data have suggested that the black population is at higher risk of developing secondary hyperparathyroidism.^12^ In the present study, only 13% of the population was black, and this did not influence the results. In analyzing the etiologies of chronic kidney disease we observed that arterial hypertension, diabetes and unknown causes were responsible for 78% of the cases. Some reports have suggested that diabetic patients show a lower incidence of secondary hyperparathyroidism.^13^ However, such evaluation was outside of the objectives of the present study.

The study population (n = 131) was divided according to two criteria. First, according to their creatinine clearance and, subsequently, by iPTH level. Measurements of iPTH by immunochemiluminometric assay showed high intra-assay reproducibility, which corroborates data from the literature suggesting the utility of this method.^14^ The present data showed that the median iPTH was significantly higher among patients with reduced renal function. The biochemical serum data did not show any difference in phosphorus levels, a parameter that has been implicated as an independent factor for increases in PTH.^15^ This was probably a consequence of the dietary control and the phosphorus binder usage by all patients in our study. The mean values for calcium and total CO_2_ were significantly lower among patients with reduced renal function, and their median alkaline phosphatase was higher.

Although statistically different, the absolute values for all the abovementioned parameters were within the normal range. It was demonstrated that patients with early chronic kidney disease, and even in more advanced phases, showed normal values for Ca and P despite the presence of elevated iPTH.^15^ In the present study, when groups were analyzed according to iPTH level, they showed similar values for albumin and estimated protein ingestion, thus suggesting good dietary control. Although the mean values for calcium and phosphorus were within the normal range, they were lower and higher, respectively, in the group with iPTH > 144 pg/ml. Alkaline phosphatase was similar for the two groups, thus suggesting that it is not useful as an independent marker for hyperparathyroidism within this population. Patients showing iPTH > 144 pg/ml presented low total CO_2_, meaning that metabolic acidosis was the only abnormal serum parameter in patients with higher levels of iPTH. This result points to the fact that, even with good dietary control, phosphorus binder usage, and normal serum levels for calcium and phosphorus, patients may present metabolic acidosis and hyperparathyroidism.

We conclude that measurement of iPTH from the early beginnings of chronic kidney disease, and also the control of metabolic acidosis, are of great importance in this population. Metabolic acidosis, which is known to correlate with renal osteodystrophy, has been shown to favor PTH actions such as increasing osteoclastic activity and reducing vitamin D levels. Additionally, it acts within the bone to promote an efflux of cations (like calcium) to the blood.^16^ This efflux leads to mineral loss from bone, thereby worsening the osteodys-trophy. Other studies^17^ have demonstrated that normalization of acidosis in chronic renal patients led to increased 1,25(OH)2D3 levels and, consequently, inhibited PTH production. These results suggest that metabolic acidosis in these patients needs to be well controlled, in order to minimize the effects of hyperparathyroidism.

There is a lack of evaluations of markers for bone disease in patients under conservative management: the majority of studies have been performed on patients under dialysis treatment. Bone alkaline phosphatase is considered to be among the non-invasive markers of bone disease. It is more sensitive and specific for renal osteodystrophy, and good correlation with iPTH has been shown.^18^ Two studies have analyzed bone alkaline phosphatase in chronic renal patients before they started dialysis. One of them evaluated children,^18^ and thus cannot be compared with results from adults. The other study,^9^ evaluated 28 patients with GFR < 26 ml/min/1.73 m^2^ who were not under dietary control or phosphorus binder usage; it showed moderate correlation between bone alkaline phosphatase and iPTH. Histological evaluations were not performed on any of these subjects. A recent study^19^ analyzing 84 patients with creatinine clearance < 5 ml/min/1.73 m^2^ showed only eight patients with hyperparathyroidism, and bone alkaline phosphatase was a good marker for detection of adynamic bone disease.

In the present study, patients with elevated iPTH levels, showed normal values for albumin, estimated protein ingestion, calcium, phosphorus and alkaline phosphatase. The only abnormality observed was a tendency towards metabolic acidosis. Although iPTH was twice its upper limit, bone alkaline phosphatase levels were normal. Comparison between iPTH and bone alkaline phosphatase showed no correlation, although good correlation between bone alkaline phosphatase and alkaline phosphatase (r = 0.69; p < 0.001) was observed.

Nine patients with iPTH > 144 pg/ml were submitted to bone biopsy. Histological analysis showed that all of them presented hyperparathyroidism abnormalities: even the patients with normal bone alkaline phosphatase. These data suggest that bone alkaline phosphatase lacks accuracy as a marker for high-turnover disease in this population. Moreover, at this stage of chronic kidney disease, it was no more sensitive or specific than alkaline phosphatase, which is cheaper and easier to evaluate. There are few data in the literature showing bone histological evaluations from patients with chronic kidney disease under conservative treatment. Solal et al.^17^ found high prevalence of osteitis fibrosa among 27 patients who were not submitted to dietary control or usage of phosphate binders. Carvalho et al.,^2^ showed high prevalence of mixed and adynamic diseases among 23 patients without specific treatment. Lafage-Proust et al.^20^ studied 16 patients under very low protein diet supplemented with ketoanalogs, and found high prevalence of normal bone, followed by low-turnover disease; however, these patients showed iPTH levels of twice its normal upper limit. Baker et al.^6^ showed, in 16 patients submitted to bone biopsies, that calcitriol usage in low doses is safe and that it improved the hyperparathyroidism lesions. Hamdy et al.^5^ studied 176 patients and found high prevalence of osteitis fibrosa. They also suggested that administration of vitamin D to this population would be beneficial. Another study^4^ on 76 patients showed that mixed renal osteodystrophy and adynamic disease was more common. The heterogeneity of the results reflects the wide spectrum of iPTH levels, the diversity of the criteria used by pathologists, local factors, dietary control, usage of vitamin D, etc. Another difficulty in comparing these studies is that creatinine clearance ranged from 60 ml/min/1.73 m^2^ to 5 ml/min/1.73 m^2^, and the study populations were not followed up using specific treatment.

In the present study, the group presenting creatinine clearance from 10 to 30 ml/min/1.73 m^2^ and iPTH > 144 pg/ml was homogenous. It was followed up by specialists and showed normal biochemical serum profiles. Even so, those submitted to bone biopsies presented abnormalities. The present results are in agreement with the suggestion by Hutchinson^1^ that iPTH levels should be maintained up to twice the normal upper limit for this population of chronic renal patients.

In summary, normal serum levels of calcium, phosphorus, alkaline phosphatase and bone alkaline phosphatase in chronic renal patients under conservative treatment do not imply normal iPTH. Metabolic acidosis was the only laboratory abnormality in this population, and this was associated with secondary hyperparathyroidism. Patients with iPTH of twice its normal value may present the histological lesions of hyper-parathyroidism.

## CONCLUSION

These data suggest the importance of early control of PTH and metabolic acidosis, in order to avoid complications related to renal bone disease.
